# Perspective: Isoflavones—Intriguing Molecules but Much Remains to Be Learned about These Soybean Constituents

**DOI:** 10.1016/j.advnut.2025.100418

**Published:** 2025-03-27

**Authors:** Mark Messina, Stephen Barnes, Kenneth DR Setchell

**Affiliations:** 1 Nutrition Science and Research, Soy Nutrition Institute Global, Washington, DC, United States; 2 Division of Pulmonary, Allergy and Critical Care Medicine, Heersink School of Medicine, University of Alabama at Birmingham, Birmingham, AL, United States; 3 Clinical Mass Spectrometry, Division of Pathology and Laboratory Medicine, Cincinnati Children’s Hospital Medical Center, Cincinnati, OH, United States

**Keywords:** isoflavones, soy, breast cancer, SERMs, intake recommendations, equol, phytoestrogens

## Abstract

Isoflavones are naturally occurring compounds found in a wide range of plants, but among commonly consumed foods are especially abundant in soybeans and foods derived from this legume. Much of the substantial amount of research conducted on soy protein and soy foods over the past 30 y is because of their isoflavone content. Research interest in isoflavones increased dramatically beginning in the early 1990s as evidence highlighted their possible role in the prevention of a wide range of cancers, including breast, prostate, and colon cancer. Recognition that isoflavones preferentially bind to estrogen receptor (ER)β in comparison with ERα provided a conceptual basis for classifying these diphenolic molecules as selective ER modulators (SERMs). Isoflavone research soon greatly expanded beyond cancer to include areas such as coronary artery disease, bone health, cognitive function, and vasomotor symptoms of menopause. Nevertheless, safety concerns about isoflavones, based primarily on the results of rodent studies and presumed estrogenic effects, also arose. However, recent work challenges the traditional view of the estrogenicity of isoflavones. Furthermore, safety concerns have largely been refuted by intervention and population studies. On the other hand, investigation of the proposed benefits of isoflavones has produced inconsistent data. The small sample size and short duration common to many intervention trials, combined with marked interindividual differences in isoflavone metabolism, likely contribute to the conflicting findings. Also, many different intervention products have been employed, which vary not only in the total amount, but also in the relative proportion of the 3 soybean isoflavones, and the form in which they are delivered (glycoside compared with aglycone). For those interested in exploring the proposed benefits of isoflavones, studies justify an intake recommendation of ∼50 mg/d, an amount provided by ∼2 servings of traditional Asian soy foods.

## Introduction

Isoflavones have been the subject of intense research over the past 30+ y as evidenced by >25,000 isoflavone-related articles indexed in PubMed. Isoflavones bind to estrogen receptors (ERs) and are therefore commonly classified as phytoestrogens. These naturally occurring compounds are present in a wide range of plants but among commonly consumed foods, are found in uniquely high amounts in soybeans [1]. Much of the considerable amount of research conducted over the past 3 decades on soy foods and soy protein is because of their isoflavone content. However, despite the voluminous amount of research, there is still an incomplete understanding of the health effects of these diphenolic soybean components, a point made clearer by the recent publication by Viscardi et al. [2]. As will be discussed, their findings shed important light on the classification of isoflavones as phytoestrogens.

With this background in mind, this review provides general information about isoflavones, a timeline of modern isoflavone research, a perspective on the efficacy and safety of isoflavones, and includes intake recommendations. For information on the possible impact of in-utero isoflavone exposure and exposure during breastfeeding, topics which are not discussed in this manuscript, the reader is referred to the reference [3].

### Isoflavone intake and content in soybeans

**FIGURE 1:**
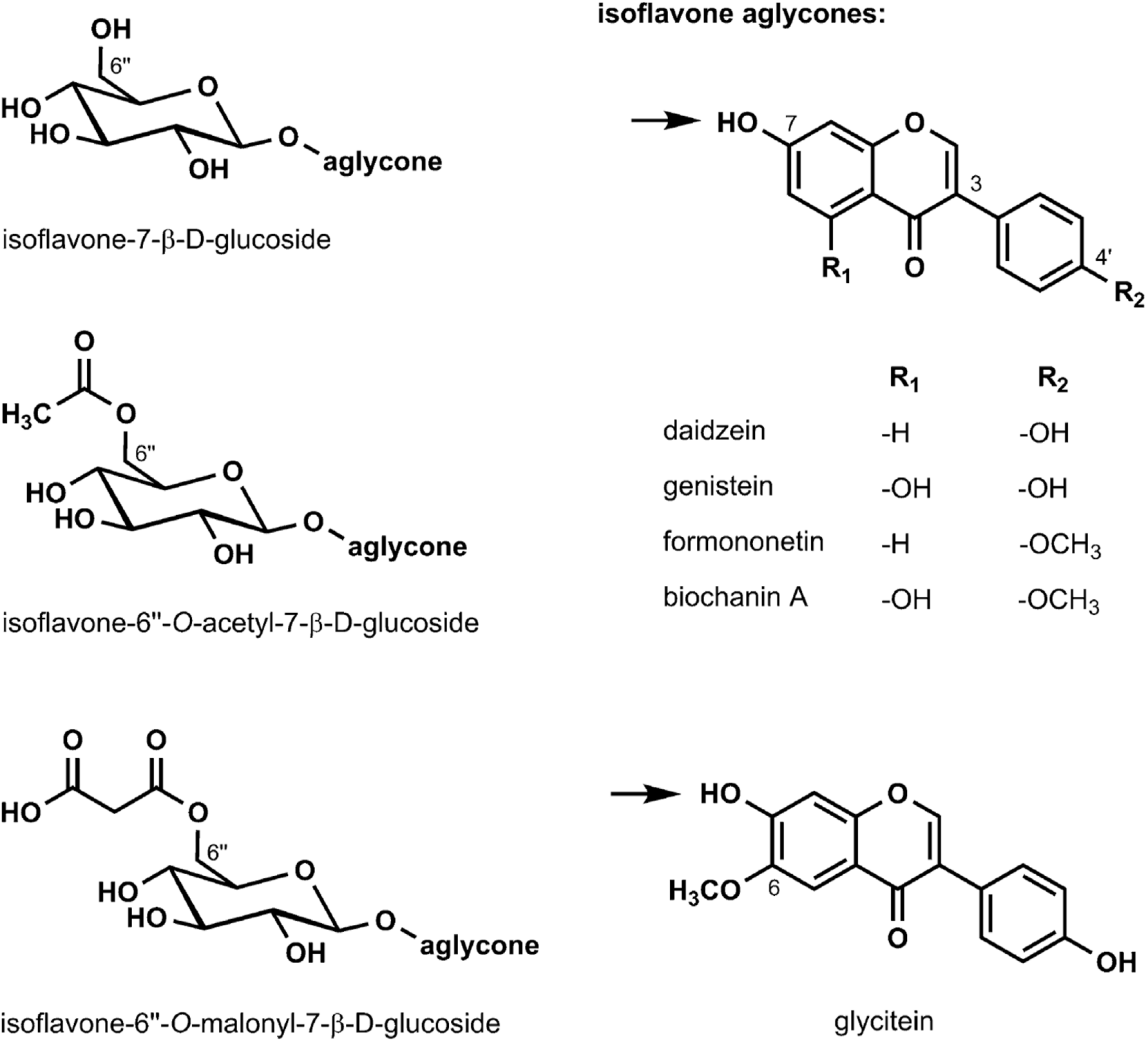
Chemical structures of isoflavones in soybeans and red clover and their glucose conjugates (left row). The 7-*O*-ß-D-glucosides of daidzein, genistein, and glycitein are daidzin, genistin, and glycitin, respectively. The arrows in the figure indicate the position of glucose conjugation. Reprinted with permission from Springer Nature. Figure appeared in *Archives of Toxicology* volume 92 Issue 9 pages 2703-2748, 2018.

The 3 isoflavones in soybeans are the β-glucosides genistin, daidzin, and glycitin, and their respective aglycones, genistein, daidzein, and glycitein [4] (Figure 1). In the soybean before processing, the glucosylated isoflavones are esterified with malonic acid at the 6-hydroxyl position of the glucose moiety. This is important because processing to make soy foods may or may not retain the ester group. For instance, for soy foods made from defatted soy flour and then toasted, the malonyl group is decarboxylated to an acetyl group. This may also apply to isolated soy protein. In fermented soy foods, the glucosyl groups are removed and modification of the isoflavone may have occurred. These different chemical forms of soy isoflavones determine whether they undergo digestion by intestinal β-glucosidases and hence their uptake from the small intestine, as well as first-pass metabolism by bacterial β-glucosidases and other microflora in the large intestine. Interestingly, consumption of a soy protein isolate rich in acetylated isoflavone glucosides is associated with a >6 h delay in the appearance of glucuronidated isoflavones in blood suggesting that absorption did not occur in the small intestine and was dependent on bacterially induced hydrolysis in the colon [5,6].

Genistein, daidzein, and glycitein (and their different chemical forms) comprise ∼50%, 40%, and 10%, respectively, of total isoflavone content, although there is considerable variation in this ratio among soybean varieties and foods produced from soybeans [4,7]. In soybeans and unfermented soy foods, isoflavones occur almost entirely as glycosides. Because the weight of the nonisoflavone part of glycosides (sugar molecule) accounts for ∼40% of the total weight, a value of 0.6 is typically used to convert glycoside into aglycone values, the biologically relevant amount. Unless otherwise indicated, throughout this manuscript, isoflavone amounts refer to the adjusted aglycone weight.

There are ∼3–4 mg isoflavones per gram of protein in traditional Asian soy foods such as tofu, soymilk made from whole soybeans, and edamame [8]. Thus, 1 serving, such as 1 cup soymilk or 3–4 oz tofu, provides ∼25 mg isoflavones. Due to losses during processing, the isoflavone content of concentrated forms of soy protein such as soy protein isolate and soy protein concentrate, which are 90% and 65% protein [9], respectively, typically ranges from 0.5 to 2 mg/g protein [4,10]. Mean daily isoflavone intake among native Japanese consuming a traditional diet ranges from ∼30 to 50 mg/d [8,[11], [12], [13]] compared with <3 mg/d in the adults living in the United States [14] and Europe [15]. Isoflavone intake in other Asian countries is typically lower than in Japan, although intake in China varies markedly among regions, with some regions such as Shanghai, equaling or exceeding Japanese intake [[16], [17], [18], [19]]. However, westernization of diets has led to a lower isoflavone intake among younger Asians [20].

### Early isoflavone observations

Genistin and daidzin were first identified in soybeans in the 1930s [21,22], although not until 1973 was the third isoflavone glycitein discovered [23]. Isoflavones first came to the attention of agricultural scientists in the 1940s when it was concluded that the infertility problems experienced by female sheep in Western Australia stemmed from an isoflavone-rich red clover the animals grazed on [[24], [25], [26], [27]]. The high content of formononetin (a precursor molecule to daidzein) in clover combined with the large amounts of red clover consumed led to the development of what was referred to as “Clover disease,” a cystic endometrium with associated fibrosis of the muscular layer of the uterus. To put this issue into perspective, in the 1940s, sheep were consuming >80 g/d of isoflavones when grazing in fields rich in *Trifolium subterraneum.* For humans to be exposed to this level of isoflavones, they would need to consume >1300 L of soymilk or 360 kg of tofu daily. Blood levels of equol, the exclusive metabolite of formononetin in sheep, reach in excess of 20 mmol/L which is markedly higher than that attainable in humans consuming soy foods [28]. In addition to formononetin, red clover also contains biochanin A. These O-methylated isoflavones are converted in vivo to daidzein and genistein, respectively, by cytochrome P450s [29].

Despite the problems noted in sheep in the 1940s, recent work does not support the fertility concerns of red clover [30]. The reason for the lack of confirmation is unclear, but the current consensus among farmers is that red clover does not adversely impact ewe reproduction. It may be that the sheep in Australia were historically consuming far higher amounts of clover than is typically the case today.

In any event, 3 decades later, the liver disease and reproductive problems in the captive cheetah in North American zoos were attributed to the feed that contained isoflavones from soy meal [31]. However, these reproductive results are unlikely to have implications for humans because cheetahs, indeed, the entire cat family, have stop codons, insertions, and deletions in the gene encoding the phase II enzyme, UGT1A6, which is responsible for glucuronidation, and hence, the usual rapid elimination of isoflavones is impaired [32]. Differences in isoflavone metabolism between rodents and humans also highlight the cautious approach needed when extrapolating the results from one species to another [33].

### Isoflavones as phytoestrogens

In the 1950s, investigators demonstrated the ability of genistein to stimulate uterine growth in immature mice, a classic test of estrogenicity. As a result of this “estrogenic” property, isoflavones were considered for use by the animal feed industry as growth promoters [[34], [35], [36]]. They continue to be investigated for their role in enhancing the well-being of livestock [[37], [38], [39]].

In the 1960s, Folman and Pope [40,41] established the relative binding affinities of soybean isoflavones for ERα, the only ER known at that time. Somewhat prophetically, their work led them to conclude that the importance of genistein “… might lie as much in its ability to antagonize the natural steroid estrogens as in its own estrogenic activity” [40]. Almost simultaneously and in agreement, Shutt [42] established that in ovariectomized mice, genistein was able to displace estradiol from the ER in reproductive tissues. Although the identification of this antiestrogenic property may have foreshadowed later thinking about isoflavones, it did not appear to stimulate research.

In 1980, Drane et al. [43] demonstrated the uterotrophic effects of isoflavones in weanling female mice and noted the increasing use of soy as a food for humans, but cautioned against extrapolating the results because of the well-known species differences in susceptibility to the effects of estrogen. Equol, which was first discovered in the urine of pregnant mares in 1932 [44] and identified in human urine in 1982 [45], was shown to be synthesized from the soy isoflavone daidzein in a segment of the population who hosts the necessary intestinal microbiota [46]. Two years later, Setchell et al. [47,48] showed for the first time that a subset of females can produce equol in response to soy consumption. Current estimates are that ∼25%–30% of non-Asians [49,50] and perhaps ∼50% of Asians [50] and Western vegetarians [51] are equol-synthesizers, although some data suggest the percentage of equol-synthesizers among Asians is lower than initially suggested [52].

In their 1984 article, Setchell et al. [47] speculated that due to their possible antiestrogenic effects, isoflavones and equol could play a role in reducing the risk of developing breast cancer, although they also warned about adverse effects on reproduction, due to the possible estrogen-like effects of isoflavones. In some respects, this hypothesized dual role of isoflavones (ER agonists and antagonists), which was also suggested by Folman and Pope [40], foreshadows interest in the concept of selective estrogen receptor modulators (SERMs) [53]. SERMs, such as the breast cancer drug tamoxifen, have estrogenic effects in some tissues but either no effects or antiestrogenic effects in other tissues [54]. However, even classifying isoflavones as SERMs may be an oversimplification because in in vitro and preclinical models, isoflavones and equol exhibit myriad other properties independent of ER binding, such as inhibiting oxidation and inflammation [55,56].

### Isoflavone research increases exponentially in the 1990s

It was the proposed breast cancer prevention effect that led to the explosion of isoflavone research that followed soon thereafter. In 1990, Barnes et al. [57] demonstrated that isoflavone-rich soy protein, but not soy protein devoid of isoflavones, inhibited the development of chemically induced mammary cancer in rats. This finding prompted the US National Cancer Institute to hold a workshop on the role of soy in cancer prevention, the findings from which led this institute to fund isoflavone research [58]. The historically low rates of breast cancer in Japan helped fuel enthusiasm for work in this area [59]. Recognition by a federal funding agency that isoflavones may have important physiological effects in humans lent considerable credence to the field.

It would be remiss not to mention that although the focus was primarily on the hormonal properties of isoflavones, it was demonstrated in 1987 that in vitro, genistein inhibits the activity of tyrosine protein kinase [60]. Not long after, it was shown that genistein also inhibits the activity of DNA topoisomerases in vitro [61,62]. Tyrosine kinases are overexpressed in cancer cells and are implicated in several steps of neoplastic development and progression [63]. DNA topoisomerases catalyze changes in the topological state of DNA [64]. These findings further increased interest in isoflavones because they provided potential mechanisms by which isoflavones could inhibit the development of a range of cancers independent of their interaction with ERs, although these in vitro effects occurred at concentrations beyond that which could likely be achieved in vivo. This latter point warrants emphasis because, although the consumption of high amounts of isoflavones (100 mg/d) produces low micromolar isoflavone concentrations—concentrations at which a range of effects are observed in vitro—in the circulation, ≥98% of the isoflavones are conjugated with glucuronic acid or sulfate, forms that are mostly biologically inactive [33].

Isoflavone research increased dramatically throughout the 1990s, as illustrated by the annual number of isoflavone-related articles indexed in PubMed (using isoflavones, genistein, daidzein, and equol as keywords), increasing from 101 in 1990, to 495 in 1995, to 768 in 1999. The topics under investigation also greatly expanded. For example, the first trial evaluating the effects of isoflavone-rich soy protein on hot flashes was published in 1995 [65]; 3 y before that Adlercreutz et al. [66] hypothesized that isoflavones might account for the relatively low incidence of menopausal symptoms reported by Japanese females.

Evaluating the clinical effects of isoflavones became easier with the advent in the mid-1990s of isoflavone supplements (tablets containing primarily isoflavones and no protein). However, the first supplement (soy germ) was produced via mechanical separation of isoflavones from the hypocotyl portion of the soybean, which has a different isoflavone profile than the remaining 90% (cotyledon). It is very low in genistein (∼10% of total isoflavone content) [67], the isoflavone thought to be the most biologically potent of the 3 soybean isoflavones based on the relative binding affinity for ERs [68]. As noted previously, all 3 isoflavones as well as the metabolite equol, preferentially bind to ERβ in comparison to ERα. Equol is an interesting molecule because it has been reported that about half of it in the circulation is in the free (unbound to plasma proteins) and biologically active form, much higher than is the case for the 3 soybean isoflavones [69].

With respect to hot flash alleviation, there continues to be disagreement about the efficacy of isoflavones. A 2012 meta-analysis of 13 randomized controlled trials (RCTs) by Taku et al. [70] found that isoflavones were efficacious but only if sufficient genistein was ingested. These authors concluded that taking 50 mg/d total isoflavones is efficacious if at least ∼20 mg that amount is genistein. The Spanish Menopause Society recently listed isoflavones as an alternative treatment for the alleviation of moderate hot flashes [71] but because of the variable findings, the North American Menopause Society does not endorse isoflavones [72]. However, in their treatment of the data, trial results were not subanalyzed according to the genistein content of the intervention product [73].

Skeletal benefits of isoflavones were reported in 1996 in rodents [74] and in postmenopausal women in 1998 [75]. In recent years, several meta-analyses of RCTs investigating the effects of isoflavones on bone turnover and/or bone mineral density (BMD) have been published [[76], [77], [78], [79]]. In 2024, a meta-analysis of 63 RCTs comparing isoflavone interventions (*n* = 4754) and placebo (*n* = 4272) showed that in postmenopausal women, isoflavones significantly improved BMD at the lumbar spine, femoral neck, and distal radius. Subgroup analysis indicated that isoflavones were efficacious when the intervention contained ≥50 mg/d genistein [76]. Despite this conclusion, it is important to recognize that of the 4 longer-term (≥2 y duration) RCTs involving isoflavone interventions in postmenopausal women [[80], [81], [82], [83]], only 1 [83] reported BMD improved. Given that many shorter-term RCTs have evaluated bone turnover, it is suggested that future clinical work in this area focus on changes in BMD over a minimum of 2 y.

Coronary benefits in monkeys were first demonstrated in 1996 [84]. These findings contributed to speculation that isoflavones could function as alternatives to conventional menopausal hormone therapy [85,86]. The cholesterol-lowering effect of soy protein was first demonstrated clinically in 1967 [87], an effect that was largely overlooked until the publication of a meta-analysis in the *New England Journal of Medicine* nearly 30 y later [88]. However, the effects of isoflavones focused on arterial health, not cholesterol levels.

In 2002, Italian researchers showed that genistein aglycone (54 mg/d) enhanced endothelial function in postmenopausal women as assessed by flow-medicated dilation (FMD) [89]. A 2010 meta-analysis of 9 RCTs, in which isoflavones in doses between 50 and 100 mg/d were provided in the form of tablets (*n* = 3) or soy protein (*n* = 6), found improved endothelial function in postmenopausal women with impaired, but not normal endothelial function at baseline [90]. A Bayesian meta-analysis of 17 RCTs published 2 y later found a modest beneficial effect of isoflavones on FMD [91]. However, a subsequently published meta-analysis did not show beneficial effects although this analysis, which included only 5 RCTs, excluded studies that intervened with isoflavones in supplement form [92].

As noted at the onset, the potential breast cancer-preventive effects of isoflavones garnered considerable research attention. Observational studies generally show that isoflavone intake is associated with a reduced breast cancer risk. For example, a meta-analysis of 24 observational studies found a statistically significant 29% reduction in risk when comparing high with low isoflavone intake, although protective effects were only observed in case-control (*n* = 17) not cohort (*n* = 7) studies [93]. However, in the dose–response meta-analysis of the cohort studies, a 10 mg/d increase in isoflavone intake was associated with either a 6.8% or 3.2% decrease in breast cancer risk, depending on the way in which the data were analyzed. In any event, clinical research in which markers of breast cancer risk, such as breast tissue density [83,[94], [95], [96], [97], [98], [99]] and breast cell proliferation [[100], [101], [102], [103], [104], [105]], have been assessed, has not provided support for the proposed breast cancer-protective effects of isoflavones.

It may be, that as was first proposed in 1995, for isoflavones to most effectively reduce breast cancer risk, consumption must occur during childhood and/or adolescence [106,107]. For obvious reasons, this hypothesis, which has gained support over the years [[108], [109], [110], [111]], is difficult to explore clinically in girls. Nevertheless, it warrants more attention given the public health significance and findings from observational studies indicating just 1 serving/d of soy foods is sufficient to reduce risk. One approach to evaluating this hypothesis may be to assess epigenetic changes and changes in the expression of cancer-related genes in girls in response to soy consumption [112,113].

### Isoflavones as putative SERMs

The discovery of ERβ in 1996 [114] and the finding in 1998 [115] that isoflavones preferentially bind to this receptor provided a molecular basis for classifying isoflavones as SERMs [54,116]. These receptors have a different tissue distribution within the body and, in general, activation of ERα and ERβ is seen as exerting proliferative and antiproliferative effects, respectively [117].

Although much of the isoflavone research focused on women, and postmenopausal women in particular, there was also interest in the role of isoflavones in reducing the risk of prostate cancer as this cancer is hormonally driven [118], and like breast cancer, historical incidence rates are low in Japan [59]. Isoflavones were shown to inhibit chemically induced prostate cancer in rats in 1997 [119] and to lower prostate-specific antigen levels in men in 2001 [120]. Interestingly, the isoflavone metabolite equol was shown to block the trophic effect of 5-α-dihydrotestosterone on ventral prostate growth and inhibit the feedback effect on plasma luteinizing hormone levels in castrated rats [121]. The sequestering of 5α--dihydrotestosterone, the most potent androgen, potentially has implications for prostate cancer prevention. However, 2 trials 2–3 y in duration failed to provide support for the efficacy of isoflavones. In 1, isoflavones (43 mg/d) had no effect on prostate cancer progression in men who underwent radical prostatectomy [122] and in the other, isoflavones (∼100 mg/d) did not affect the time to the development of prostate cancer in men with high-grade prostatic intraepithelial neoplasia, a precancerous lesion [123]. On the other hand, a meta-analysis of 22 observational studies comprising 1,409,213 men showed that isoflavone intake was associated with a reduced risk of prostate cancer, especially in cases of localized or low-grade prostate cancer [124]. But importantly, when the data were subanalyzed according to ethnicity, no effect was noted among Japanese men, the group with the highest intake among the various ethnicities evaluated. This finding serves to highlight the conclusions of a recently published perspective that epidemiological findings based on low-soy intake populations have limited ability to provide insight into the health effects of isoflavones [125]. In high-soy-consuming populations, upper-intake groups typically consume ≥50 mg/d isoflavones [125].

The impact of isoflavones on cognition [[126], [127], [128], [129], [130], [131], [132], [133]] and the condition of the skin, including wrinkles [134,135], has also attracted attention, although to a lesser extent than the previously discussed research areas. Clinical research into the cognitive effects of isoflavones began in the early 2000s [126,128]. In 2020, a meta-analysis of 16 RCTs involving 1386 mostly postmenopausal women with a mean age of 60 y showed that isoflavones improved overall cognitive function but of the various individual cognitive function domains examined, there was a statistically significant improvement only in memory [136]. The median duration of the intervention was 17 wk (range, 6–130 wk) and the dose of isoflavones ranged from 60 to 160 mg/d.

With respect to skin, many of the RCTs suffer from design limitations, although in a small but well-designed recently published 6-mo study, Rizzo et al. [137] found that in comparison with casein (30 g/d), isoflavone-rich soy protein that provided 50 mg isoflavones reduced wrinkle severity and pigment intensity, and improved skin hydration. Additional longer-term studies aimed at establishing dose–response relationships, effects in females with darker skin, and whether changes revert to baseline values on isoflavone discontinuation, are warranted.

### Challenges to the “estrogenic” nature of isoflavones

For the most part, with respect to both benefits (except for breast cancer prevention) and safety, research on isoflavones was driven by their ER agonist properties, properties that were assumed to be most evident in postmenopausal women. However, the newly published systematic review and meta-analysis of 40 RCTs and 52 trial comparisons by Viscardi et al. [2], calls into question the traditional view of the estrogenicity of isoflavones. In brief, these authors found that in postmenopausal women, isoflavones (median trial duration, 24 wk; median isoflavone dose, 75 mg/d) were without significant effect on endometrial thickness, vaginal maturation index, and circulating levels of follicle-stimulating hormone and estradiol, all metrics that are affected by the hormone estrogen. This lack of effect does not rule out isoflavones from exerting estrogen-like effects on tissues and endpoints not examined by Viscardi et al. [2], and in populations other than postmenopausal women, but it certainly reinforces the notion that isoflavones likely differ from estradiol beyond differences in relative binding affinity for, and transactivation of, ERs. In some respects, the results by Viscardi et al. [2] are not surprising because although isoflavones bind to ERs, it has long been known that they show distinct and significant differences in properties from endogenous steroidogenic estrogens. In fact, it is not easy to identify trials in which isoflavones demonstrate clear estrogenic or antiestrogenic effects.

### Despite proposed benefits, isoflavones have been mired in controversy

The clearest examples of possible adverse effects of isoflavones based on their estrogenicity may be case reports, each describing a single male that experienced feminizing effects (for example, gynecomastia, loss of libido) allegedly due to soy consumption [[138], [139], [140], [141]]. These reports may have contributed to the coining and use of the denigrating term “soy boy” [142]. However, in 3 of the 4 cases, feminizing effects occurred in response to isoflavone intakes 8–10 times higher than typical Japanese intake and likely in the context of unbalanced and nutrient-deficient diets. Information on isoflavone intake was not provided in the most recent case report, although homemade soymilk consumption was reported to be 0.5–1.0 L daily [141]. However, because gynecomastia was still present despite soymilk consumption being discontinued for the 2 prior years, it is unclear that isoflavones played a role in the occurrence of this condition.

Furthermore, and more importantly, in response to isoflavone intakes (≤100 mg/d) that can realistically be consumed via the consumption of soy foods, results from RCTs involving healthy men show no effects on circulating estrogen or testosterone levels [143], gynecomastia [123,144], or sperm or semen parameters [[145], [146], [147]]. The lack of effect on testosterone and estrogen is based on a meta-analysis that included 41 RCTs [143]. Total testosterone and free testosterone levels were measured in 1753 and 752 men, respectively, and estradiol and estrone levels were measured in 1000 and 239 men, respectively. Subanalysis revealed that neither study duration (≤12 wk compared with >12 wk) nor isoflavone dose (<75 mg/d compared with ≥75 mg/d) affected the impact of isoflavone exposure on these hormone concentrations.

Intriguingly, 2 case reports published 2 decades apart describe a single male with low sperm concentrations that became normalized and that led to pregnancy in their mates after the consumption of 80 mg/d isoflavones for 6 mo [148,149]. Also, with respect to feminization, soy protein containing isoflavones has been shown to promote gains in muscle mass and strength in those engaged in resistance exercise training similarly to animal protein [150].

Claims of male feminization are certainly not the only concerns based on presumed estrogen-like effects that have been raised about isoflavones. Most notable is the concern that postdiagnosis soy intake could worsen the outcomes of patients with breast cancer [151,152]. This concern is based primarily on the results of studies in rodents that began to be published in the late 1990s [151] and continued for 2 decades [152,153]. However, this adverse effect has not been supported in observational studies that evaluated recurrence and/or survival [[154], [155], [156], [157]] or clinical studies that evaluated markers of breast cancer risk [3,158]. In fact, in 2022, the American Cancer Society concluded that postdiagnosis soy intake may reduce recurrence and improve survival [159] and in 2023, the World Cancer Research Fund International (Global Cancer Update Programme) identified soy intake as 1 of 5 factors that may improve the survival of patients with breast cancer. Nevertheless, more research is needed on the impact of isoflavones in women while under treatment for ER-positive breast cancer.

As noted, most safety concerns are based on the presumed estrogenic effects of isoflavones, but this is not the case for claims that isoflavones impair thyroid function, which arose in earnest in the late 1990s based on in vitro and rat data [160,161]. However, a meta-analysis of 18 RCTs published in 2019 showed that neither soy nor isoflavone intake (doses ranging from 40 to 200 mg/d) affects circulating levels of free thyroxine or free triiodothyronine [162]. In healthy adults, among the hundreds of clinical trials conducted, it is difficult to identify any significant body of research that challenges the safety of isoflavones when consumed at levels similar to those of native Japanese consuming a traditional diet.

### Challenges to the “anti-estrogenic” nature of isoflavones

Like the proposed estrogenic effects, antiestrogenic effects have also been difficult to identify. For example, although tamoxifen and aromatase inhibitors reduce mammographic density [163] and breast cell proliferation [164,165], no such effects have been observed in numerous RCTs in response to isoflavone intakes generally ranging from ∼40 to 100 mg/d, provided by soy protein or tablets [98,104].

One example of an ER antagonistic effect may be a 6-mo trial by Bitto et al. [166], who reported that genistein aglycone (54 mg/d) improved symptoms of endometrial hyperplasia in premenopausal females to the same extent as the synthetic progestin norethisterone acetate. However, in contrast, Murray et al. [167] concluded that consuming soy protein isolate daily that provided 120 mg isoflavones did not prevent estradiol-induced endometrial hyperplasia in postmenopausal women although there was an inhibitory effect on endometrial thickness. A possible antiestrogenic effect based on changes in serum complement (C)3 levels in premenopausal women was reported by Qin et al. [168], but only in response to a low (40 mg/d) and not high (140 mg/d) isoflavone dose.

### Factors possibly contributing to the inconsistent isoflavone data

The small trial sample size common to diet/nutrition studies is likely 1 factor contributing to the inconsistent data as are the different intervention durations. Also, a wide range of isoflavone doses and products have been employed in intervention studies. Trials in which isoflavone supplements have been used vary markedly in the ratio of the 3 soybean isoflavones. Because each isoflavone is a different chemical entity, products containing different isoflavone concentration ratios may have differing effects, as was demonstrated in the case of hot flash alleviation [70]. In this case, genistein-rich supplements were shown to be more efficacious than supplements low in this isoflavone.

As noted, in soybeans and unfermented soy foods, isoflavones occur almost exclusively as glycosides. These conjugated forms are not bioavailable or biologically active and require hydrolysis to permit absorption and activation [169]. Consuming isoflavones in aglycone form, thereby eliminating the first digestive step of hydrolyzing the glucose moiety, may lead to higher Cmax levels [[170], [171], [172], [173]], which in turn might enhance efficacy even if total isoflavone absorption is not affected [[174], [175], [176], [177], [178], [179]]. The importance of eliminating this step in digestion is supported by the effects of 3 clinical trials in which the intervention product was a food containing predominantly isoflavone aglycones [[180], [181], [182]]. In alignment, 1 research group intervening with isolated genistein in aglycone form has a track record of publishing RCTs in which an impressive array of benefits has been reported [83,[183], [184], [185], [186]]. Most intervention trials have employed soy foods or supplements containing isoflavones predominantly in glycoside form.

If isoflavones in aglycone form exert effects superior to those in glycoside form, then fermented soy foods may have advantages over unfermented soy foods (the primary form in which soy is consumed worldwide). To varying degrees, depending on the food and duration, fermentation leads to isoflavone glycosides being converted to aglycones [4,[187], [188], [189]]. However, support for the beneficial effects of fermented over unfermented soy foods is not evident from the relatively limited epidemiological literature. In any event, intervention studies that directly compare mixed isoflavones with isolated genistein, and that compare the aglycone genistein with its glycoside genistin, would make an important contribution to the literature. In the case of daidzein, it has been suggested that ingestion of the aglycone form may lead to greater equol synthesis [190]. Comparing equol synthesis in response to daidzein compared with daidzin is therefore also of interest.

Of all the various factors potentially affecting efficacy, interindividual differences in isoflavone metabolism may be the most important one contributing to the inconsistent data. Setchell et al. [28] proposed in 2002 that equol-synthesizers are more likely to benefit from isoflavone intake than equol-nonsynthesizers. Epidemiological support for this hypothesis continues to accumulate although clinical data are mostly lacking [[191], [192], [193], [194], [195]]. However, often underappreciated is that microbial metabolism of isoflavones markedly differs among individuals beyond equol synthesizing ability [[196], [197], [198]]. Future intervention studies should be large enough to subanalyze the results according to metabotypes [196] to allow for the possible identification of isoflavone “responders” and “non-responders.” As an example, Kwak et al. [199] found that in premenopausal females in response to the consumption of an isoflavone extract, serum concentrations of the bone formation marker osteocalcin increased in high urinary genistein excretors, but not in the low genistein excretors or the placebo group.

To varying degrees, understanding of the health effects of isoflavones comes from observational studies in which soy food and/or isoflavone intake is assessed and correlated with health status and outcomes. However, as already alluded to, it is highly unlikely that associations or lack thereof, noted between isoflavone intake and health outcomes in studies of low-soy-intake populations have a causal basis because intake is simply too low to expect a biological effect to be observed [125]. In intervention studies, the dose of isoflavones typically employed is between 40 and 100 mg/d, whereas in low-soy-intake cohorts, such as those conducted among the general population in Europe and the United States, isoflavone intake in the highest intake group is typically 1–3 mg/d [125]. Even in high-soy-intake population studies, when the primary change in risk (for example, hazard ratio) occurs when comparing the second intake quartile or quintile with the first, the results should be viewed cautiously because intake may still be too low to reasonably expect a biological effect to be evident [125].

### Isoflavone intake recommendations

Save dietary supplements, which are widely available, the only dietary approach to increasing isoflavone intake is to consume soy foods. It is important to recognize that, like all foods, they contain many biologically active components, both nutrients and non-nutrients [200]. For example, as already mentioned, soy protein has been shown to directly lower blood cholesterol levels [201,202]. Also, the blood pressure-lowering effect of soymilk recently identified by Erlich et al. [203] is unlikely to be the result of its isoflavone content. Many soy foods provide both omega-6 and omega-3 essential fatty acids, and some also provide fiber and oligosaccharides [204]. Therefore, soy foods should not be equated with isoflavones, and neither should isoflavones be equated with estrogen. For those interested in exploring the proposed benefits of isoflavones, a reasonable intake goal is 50 mg/d, an amount provided by ∼2 servings of traditional Asian soy foods. Among the clinical and observational studies reporting benefits, 50 mg/d appears to meet the efficacious threshold [125].

Ideally, isoflavone intake would come primarily from soy foods because of the many nutrients and bioactive in soy foods that are not present in supplements (tablets). Also, incorporating soy foods into the diet may result in less healthful foods being displaced, which is an additional indirect benefit. However, for individuals primarily interested in isoflavones who are unable to consume 2 serving/d of soy foods, supplements are a viable option because the absorption and metabolism of isoflavones from supplements are similar to that from soy foods [33,205].

## Author contributions

The authors’ responsibilities were as follows – All authors participated equally in the writing of this manuscript and read and approved the final version.

## Funding

The authors reported no funding received for this study.

## Conflict of interest

MM reports a relationship with Soy Nutrition Institute Global that includes: employment. SB reports a relationship with BileOmix Inc that includes: consulting or advisory and equity or stocks. KDRS reports a relationship with Asklepion Pharmaceuticals, Baltimore that includes: equity or stocks. KDRS reports a relationship with Aliveris s.r.l, Italy that includes: equity or stocks. KDRS reports a relationship with Mirum Pharmaceuticals, California that includes: consulting or advisory. All other authors report no conflicts of interest.
